# Competition and price among brand-name drugs in the same class: A systematic review of the evidence

**DOI:** 10.1371/journal.pmed.1002872

**Published:** 2019-07-30

**Authors:** Ameet Sarpatwari, Jonathan DiBello, Marie Zakarian, Mehdi Najafzadeh, Aaron S. Kesselheim

**Affiliations:** Program On Regulation, Therapeutics, And Law (PORTAL), Division of Pharmacoepidemiology and Pharmacoeconomics, Department of Medicine, Brigham and Women’s Hospital and Harvard Medical School, Boston, Massachusetts, United States of America; York University, CANADA

## Abstract

**Background:**

Some experts have proposed combating rising drug prices by promoting brand–brand competition, a situation that is supposed to arise when multiple US Food and Drug Administration (FDA)-approved brand-name products in the same class are indicated for the same condition. However, numerous reports exist of price increases following the introduction of brand-name competition, suggesting that it may not be effective. We performed a systematic literature review of the peer-reviewed health policy and economics literature to better understand the interplay between new drug entry and intraclass drug prices.

**Methods and findings:**

We searched PubMed and EconLit for original studies on brand–brand competition in the US market published in English between January 1990 and April 2019. We performed a qualitative synthesis of each study’s data, recording its primary objective, methodology, and results. We found 10 empirical investigations, with 1 study each on antihypertensives, anti-infectives, central nervous system stimulants for attention deficit/hyperactivity disorder, disease-modifying therapies for multiple sclerosis, histamine-2 (H2) blockers, and tumor necrosis factor (TNF) inhibitors; 2 studies on cancer medications; and 2 studies on all marketed or new drugs. None of the studies reported that brand–brand competition lowers list prices of existing drugs within a class. The findings of 2 studies suggest that such competition may help restrain how new drug prices are set. Other studies found evidence that brand–brand competition was mediated by the relative quality of competing drugs and the extent to which they are marketed, with safer or more effective new drugs and greater marketing associated with higher intraclass list prices. Our investigation was limited by the studies’ use of list rather than net prices and the age of some of the data.

**Conclusions:**

Our findings suggest that policies to promote brand–brand competition in the US pharmaceutical market, such as accelerating approval of non-first-in-class drugs, will likely not result in lower drug list prices absent additional structural reforms.

## Abstract 2

Ameet Sarpatwari and colleagues assess market competition among brand-name drugs with similar therapeutic effects.

## Author summary

**Why was this study done?:**

- US prescription drug spending has increased sharply over the last decade, with higher launch prices of new brand-name drugs and routine price increases on older brand-name drugs.
- Promoting greater “brand–brand” competition, which occurs between brand-name drugs indicated for the same condition, has been proposed to address high drug prices.
- Many examples exist of price increases following the introduction of brand-name competition, casting doubt on the effectiveness of brand–brand competition in the pharmaceutical market.

**What did the researchers do and find?:**

- We systematically reviewed published research on brand–brand competition and found 10 studies evaluating a wide range of drug classes.
- None of the 10 studies found that brand–brand competition lowers the published (“list”) price of existing brand-name drugs within a class.
- We found that brand–brand competition may help restrain launch list prices and that the effect of brand–brand competition may be mediated by the relative drug quality and marketing, with safer or more effective new drugs and greater marketing associated with higher intraclass list prices.

**What do these findings mean?:**

- Barriers to brand–brand competition having a greater impact on list prices include the following: physicians not prescribing the most cost-effective treatments due to lack of knowledge, constraints on payers’ abilities to set one manufacturer against another in drug price negotiations, and the misdirected incentive for pharmacy benefits managers (PBMs) to accept high list prices of brand-name drugs.
- Greater brand–brand competition alone will likely not lower list prices of brand-name drugs.

## Introduction

Prescription drug spending has risen sharply in the US over the last decade [1]. A 2018 report by the Department of Health and Human Services (DHHS) Office of Inspector General found that net spending on brand-name drugs in Medicare Part D—the prescription drug benefit program for seniors—increased 62% from 2011 to 2015, despite a 17% decrease in the number of prescriptions of these products over the same period [2]. One driver of this growth has been the introduction of novel products with high launch prices. In 2017, the median annual list price of a new cancer medication was $160,000, compared to $101,000 in 2013 [3]. Another major contributor has been routine price increases for existing products, which accounted for about 60% of the increase in US revenues for the 45 top-selling drugs between 2014 and 2017 [4]. Without intervention, the Centers for Medicare and Medicaid Services (CMS) Office of the Actuary projected that net US spending on prescription drugs will increase faster than any other major healthcare good or service over the next decade [5].

“Brand–brand” competition, which occurs between brand-name products that are indicated for the same condition and may have the same mechanism of action, has been offered as a possible policy solution to alter this trajectory [6]. After the US Food and Drug Administration (FDA) approves a drug with a novel mechanism of action to treat a particular disease (a first-in-class drug), other drugs developed by different manufacturers may emerge. In some cases, the later-arriving manufacturer was developing its drug concurrently with the first-to-market manufacturer, as with Amgen and the cholesterol-lowering proprotein convertase subtilsin-kexin type 9 (PCSK9) inhibitor evolocumab (Repatha), which was approved 1 month after another PCSK9 inhibitor alirocumab (Praluent) manufactured by Sanofi and Regeneron in 2015 [7]. In other cases, the later-arriving manufacturer observes a market opportunity and purposefully synthesizes a “me-too” version of the original product. In 2009, for example, the FDA approved the ninth 3-hydroxy-3-methyl-glutaryl coenzyme-A reductase inhibitor (“statin”) to lower cholesterol [8]. In March 2018, the FDA Commissioner proposed accelerating the agency’s approval process for non-first-in-class drugs on the assumption that greater brand–brand competition would lower drug prices [9].

Although some examples of price lowering have been observed in the US market—most notably among the new direct-acting antiviral drugs treating hepatitis C virus infection [10]—prices of existing brand-name drugs have also risen following the introduction of brand-name competition [11,12]. For example, FDA approval and subsequent widespread availability of dasatinib (Sprycel) and nilotinib (Tasigna) for the treatment of chronic myeloid leukemia (CML) had no effect on the list price of imatinib (Gleevec), an older CML treatment; instead, list prices for all 3 drugs increased steadily between 2007 and 2014 [13]. This outcome stands in contrast to “generic competition” between different manufacturers of the same drug, which occurs when a brand-name drug loses market exclusivity, and reduces the price of a drug on average 60% when 3 generic manufacturers enter the market [14].

To better understand the economic impact of brand–brand competition, we reviewed the peer-reviewed literature for studies of how new drug market entry affects prices of drugs within the same class that treat the same indications.

## Methods

### Study design and data sources

We conducted a systematic literature review using PubMed, a National Center for Biotechnology Information database of biomedical and life sciences articles, and EconLit, an academic database of economic articles produced by the American Economic Association. This study was reported according to the Preferred Reporting Items for Systematic Reviews and Meta-Analysis (PRISMA; S1 Table), and its full protocol is available in S1 Text.

### Article selection

In February 2018, we searched PubMed using the medical subject headings (MeSHs) “‘Drug Costs’ AND (‘Economics, Pharmaceutical’ OR ‘Economic Competition’) AND ‘United States,’” and EconLit using the terms “‘Drug’ AND ‘Price’ AND (‘Competition’ OR ‘Determinants’ OR ‘Factors’) AND (‘United States’ OR ‘US’)”. Different search terms were used for each database because MeSH term indexing was not available in EconLit. In both databases, we restricted our search to original investigations published in English after 1990. Identified abstracts were reviewed independently by 2 team members (JD and MZ) and used to exclude articles. Exclusion criteria included articles that did not focus on prescription drug pricing or the US market (given its unique pharmaceutical pricing dynamics), articles that focused on the effects of generic competition, cost-effectiveness studies, news stories, and opinion pieces. Inconsistent scoring was resolved by a third reviewer (AS), who examined the full text of the article. We separately identified 1 qualifying study not captured by our search terms due its atypical indexing in PubMed (despite being an original investigation of the cost of disease-modifying drugs for multiple sclerosis, it was not indexed under the MeSH “Drug Costs”) and included it in our analysis. Given the time that had passed between the initial search and publication, we conducted a follow-up search for articles published between February 2018 and April 2019. We used the same search terms for EconLit but used the non-MeSH terms "(‘Drug Costs’ OR ‘Drug Prices’ OR ‘Cost Changes’ OR ‘Price Changes’) AND (‘Competition’ OR ‘Competitors’)” for PubMed to account for potential lag between publication and MeSH term indexing.

### Data synthesis and analysis

For each original investigation, we performed a qualitative synthesis of the data. We recorded the primary objective and time period of the study. We summarized the methodology employed, including the drugs assessed, the data sources used, and the measurement of price (e.g., average sales price) used in the study. We also recorded the results and our interpretation of the findings, focusing on how market entry impacted prices of existing products in the class, how existing products in the class affected launch prices, and what variables, if any, may have modified the effect of brand–brand competition on drug prices. This work was performed by 1 team member (JD or MZ) and was reviewed by 2 others (AS and MN). Given the number and scope of the investigations identified, a quantitative synthesis of the evidence would not have been appropriate or informative.

## Results

### Taxonomy of identified investigations

**Fig 1:**
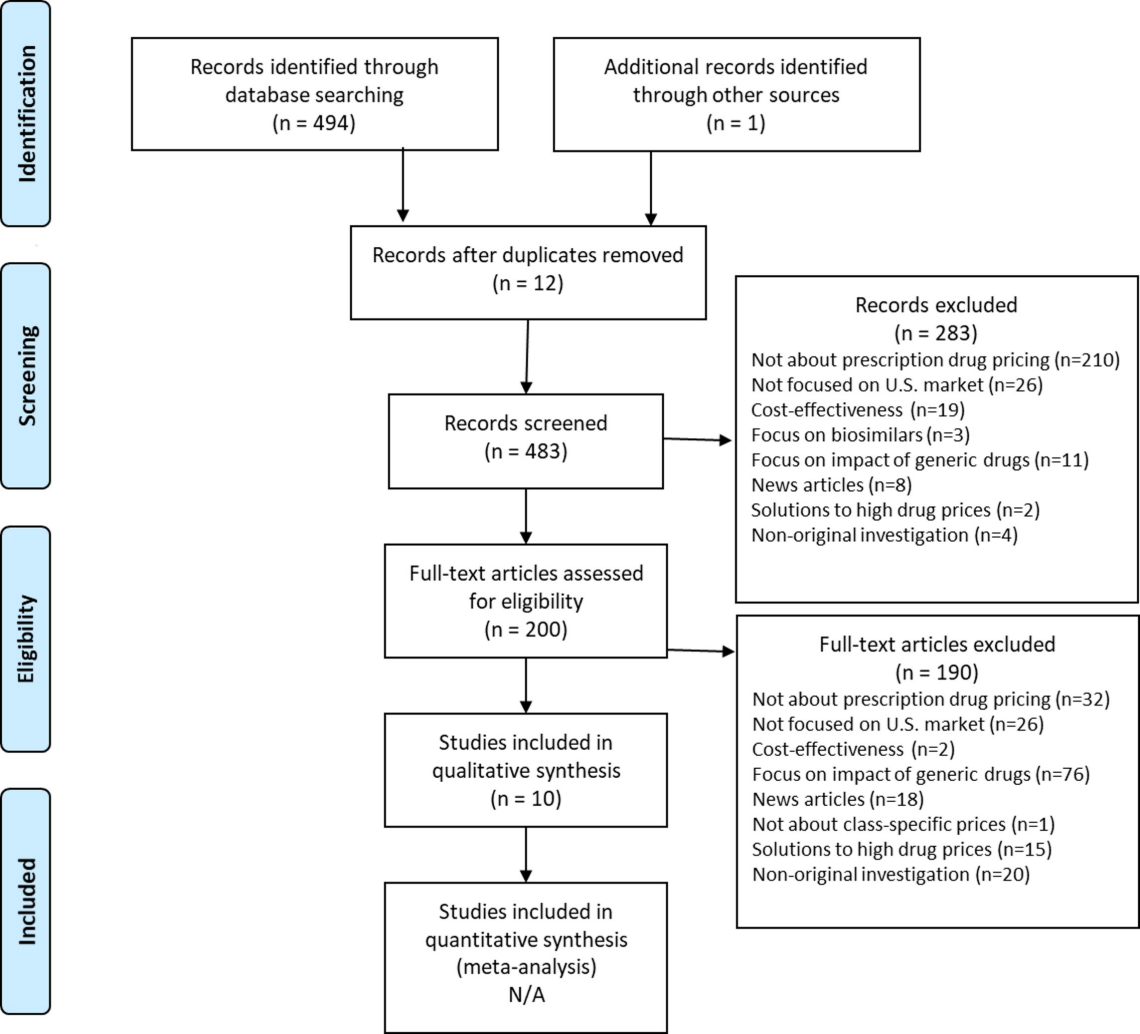
PRISMA flow-chart. N/A, not applicable; PRISMA, Preferred Reporting Items for Systematic Reviews and Meta-Analysis.

Our search yielded 483 articles, 473 of which were excluded as being not about drug pricing (*n =* 242), not focused on the US market (*n =* 52), focused on generic competition (*n =* 87), news reports (*n =* 26), cost-effectiveness studies (*n =* 21), policy analyses of solutions to high drug prices (17), nonoriginal investigations (*n =* 27), and not about class-specific prices (*n =* 1) (Fig 1). These exclusions left 10 original investigations in the final sample.

The publication dates of these studies ranged from 1994 to 2019, while the assessment periods spanned 1977 to 2016 (Table 1). Two studies evaluated all marketed or new drugs [15–16]. The other 8 were drug specific, covering histamine-2 (H2) blockers [17], antihypertensives 18], anti-infectives [19], central nervous system stimulants for attention deficit/hyperactivity disorder [20], disease-modifying therapies for multiple sclerosis [21], tumor necrosis factor (TNF) inhibitors [22], and cancer medications [23,24]. Six studies published descriptive data on drug prices over time [17,20–24].

**Table 1:** Studies included in the systematic review.

Author (Year)	Objective	Data Sources	Price Used	Principle Findings
Berndt and colleagues (1994)	To explain the growth and composition of the H2 antagonist market between 1977 and 1993	IMS sales data	Price per patient-day based on average retail price per unit	Prices of the first (cimetidine) and second (ranitidine) drugs to market increased 44% and 13% over the study period
		IMS Personal Selling Audit		The launch price of ranitidine was 56% higher than the price of cimeditine, possibly owing to a more favorable side-effect profile
				Marketing was market expanding, but the effect diminished with more products
Lu and Comanor (1998)	To assess how prices of 144 brand-name drugs approved between 1978 and 1987 were set relative to existing substitutes, and how these prices changed over time	Red Book	Treatment price based on average wholesale price per unit (calculated differently for systemic drugs used in acute nonemergency settings and for chronic conditions, topical agents, and drugs used in emergencies)	The number of brand-name substitutes was associated with lower launch prices
		FDA new product grades (A = important therapeutic gain, B = modest therapeutic gain, C = little or no therapeutic gain)		Grade A and B new drugs were priced on average 77% and 79% higher than brand-name drugs within the same class
				Over an 8-year period on the market, prices for grade A and B new drugs decreased 14% and 12%; prices for grade C drugs increased 30%
Rizzo (1999)	To assess how marketing affected competition among antihypertensive drugs between 1988 and 1993	PriceProbe, a price analysis software package from First DataBank	Treatment price based on average wholesale price per unit treatment costs (calculated assuming 100 days of therapy on most common formulation)	Marketing lowered price sensitivity
		Physicians’ Desk Reference		Marketing was market expanding and did not have a large effect on brand substitution
Wiggins and Maness (2004)	To evaluate the “price-N” relationship for anti-infective drugs between 1984 and 1990	IMS sales data	Average retail price per unit	An increase in the number of “related” drugs within the class was associated with a modest, nonsignificant decrease in drug price
Bokhari and Fournier (2013)	To assess the welfare impact of the new drug entry in the attention deficit/hyperactivity disorder market from 1999 to 2003	NDCHealth Source Territory Manager data	Average retail price per unit	Prices of most (6 of 7) marketed brand-name drugs at study initiation did not fall following the introduction of me-too drugs, but several generic drugs were already available
De Frutos and colleagues (2013)	To evaluate how drug quality differences shaped manufacturer marketing strategies between 1994 and 2003	IMS sales data	Average retail price per unit	Better quality drugs were more heavily marketed
		IMS Personal Selling Audit		
		TNS Media		Marketing was associated with higher class-wide prices
		FDA Orange Book		
Hartung and colleagues (2015)	To examine the prices of disease-modifying therapies for multiple sclerosis between 1993 and 2002 and the impact of new drug entry on these prices	First DataBank	Treatment price based on average wholesale price per unit (calculated for Medicaid assuming a 12% discount)	Prices of first-generation drugs increased from $8,000–$10,000 to approximately $60,000 per year
				Prices of new drugs were 25%–60% higher than those of existing drugs
Howard and colleagues (2015)	To assess trends in the launch prices of 58 cancer drugs approved between 1995 and 2013	CMS Average Sales Price files	Episode treatment price based on per-person cost to Medicare	There was a strong correlation (0.9) between drug pricing and incremental survival benefits
		Drugs@FDA website		After controlling for survival benefits and inflation, average launch price of cancer drugs increased about 10% per year
Gordon and colleagues (2018)	To measure the price trajectories of 24 injectable cancer drugs approved between 1996 and 2012 and examine the influence of market structure on price changes	CMS Average Sales Price files	Mean monthly cost using average retail price per unit	The mean annual increase in the monthly cost of the drugs was 3.73%, while the mean annual health-inflation rate was 1.15%
		CenterWatch		In 3 regression models, new brand-name competitors were not significantly associated with price changes of existing products
San-Juan-Rodriguez and colleagues (2019)	To assess how prices of existing TNF inhibitors changed in response to market entry of new TNF inhibitors	First DataBank	Annual cost based on wholesale acquisition cost per unit (adjusted for class-specific rebates reported for Medicare Part D)	The mean annual cost of 3 TNF inhibitors increased 144% between April 2009 and December 2016, over which time 3 new TNF inhibitors entered the market; a 33% increase was expected in the absence of these new products
		Medicare	Gross and out-of-pocket annual costs (adjusted for class-specific rebates in Medicare Part D)	Medicare Part D spending on TNF inhibitors mirrored annual costs, whereas out-of-pocket costs under the program were more stable

**Abbreviations:** CMS, Centers for Medicare and Medicaid Services; FDA, Food and Drug Administration; H2, histamine-2; TNF, tumor necrosis factor

A variety of drug price measures were employed. Three studies used average retail price per unit [16,19,20], 1 study used average retail price per unit to calculate price per patient-day [17], and 1 study used average retail price per unit to calculate price per patient-month [24]. Three studies calculated treatment price using average wholesale price per unit [15,18,21]. One study calculated annual cost using wholesale acquisition cost per unit (and also calculated annual gross and out-of-pocket costs in Medicare Part D) [22], and 1 study calculated treatment price using per-patient cost to the Medicare program [21]. Only the investigation by San-Juan-Rodriguez and colleagues [22] tried to account for drug-specific rebates from pharmaceutical manufacturers that are customarily received by payers or pharmacy benefits managers (PBMs), the entities payers contract to administer the pharmaceutical component of their plans.

### Impact of new brand-name entry on intraclass drug prices

The studies in our review did not find a price-lowering effect of new drug entry on intraclass brand-name products. Examining the H2 blocker market from 1977 to 1993—when no generics were present—Berndt and colleagues found that the average price per patient-day of the first-in-class drug cimetidine (Tagamet) fell from about $1 to $0.80 before facing competition [17]. Introduction of ranitidine (Zantac) at $1.25 per patient-day in 1983, however, did not depress the price of cimetidine further. Instead, prices of both drugs increased over time, with a faster rate of increase observed for cimetidine. This upward trajectory persisted with market entry of famotidine (Pepcid) in 1986 and nizatidine (Axid) in 1988. By the end of the study period, the average price per patient-day of cimetidine had increased 44% to $1.44, while the average price per patient-day of ranitidine had increased 13% to $1.41.

Studying the anti-infective market from 1984 to 1990, Wiggins and Maness failed to detect an association between a drug’s price and the number of existing “related” products [19]. The nonsignificant effect was far smaller than that observed for “brand-generic” competition, which occurs when interchangeable generic versions of brand-name drugs made by different manufacturers emerge after market exclusivity expires. However, the investigators did not focus on the impact of new drug entry, and their category of related products may have included generic and brand-name versions of intraclass competitors.

Bokhari and Fournier assessed the market for attention deficit/hyperactivity disorder stimulants between 1993 and 2003 [20]. Despite the introduction of 8 new brand-name drugs over this period, the average retail price of only 1 of the 7 brand-name drugs available at the start of the study period decreased. Complicating the interpretation of the analysis, however, the market also featured generic versions of 2 drugs within the class (immediate- and extended-release methylphenidate and pemoline) at study initiation.

Covering the period 1993 to 2013, Hartung and colleagues evaluated drugs for multiple sclerosis [21]. The annual treatment price of the first-generation agents interferon-β-1b (Betaseron), interferon-β-1a (Avonex), and glatiramer (Copaxone) increased from $8,292–$11,532 (initial range) to $59,158–$61,529 (ending range) over this time notwithstanding the market entry of interferon-β-1a (Rebif) in 2002, natalizumab (Tysabri) in 2004, interferon-β-1b (Extavia) in 2009, fingolimod (Gilenya) in 2010, teriflunomide (Aubagio) in 2012, and dimethyl fumarate (Tecfidera) in 2013. Following the introduction of interferon-β-1a (Rebif) and the reintroduction of natalizumab in 2006 (after its withdrawal for safety concerns).

Gordon and colleagues assessed 24 injectable cancer drugs approved between 1996 and 2012 [24]. The mean annual increase in the monthly cost of these drugs was 3.7%, more than double the mean annual health-inflation rate (1.2%). Only 1 drug, ziv-aflibercept (Zaltrap), experienced a price decline. In multivariate modeling, the introduction of new brand-name competitors was not associated with price changes of existing drugs on the market.

Finally, San-Juan-Rodriguez and colleagues evaluated the price changes of TNF inhibitors between 2006 and 2016 [22]. The investigators found that the mean annual cost of the 3 TNF inhibitors approved prior to 2009—adalimumab (Humira), etanercept (Enbrel), and infliximab (Remicade)—increased 144% between April 2009 and December 2016 despite the market entry of 3 new TNF inhibitors: golimumab (Aria), certolizumab pegol (Cimzia), and intravenous golimumab (Simponi Aria). An interrupted time-series analysis revealed that this increase was over 4-fold greater than what would have been expected if the new products were not introduced and secular trends had continued. Although the gross cost of the treatments under Medicare Part D experienced a similar trajectory, the annual out-of-pocket costs by patients in the program remained relatively stable.

### Impact of intraclass brand-name drugs on new drug launch prices

While new drug entry was not observed to lower intraclass brand-name drug prices, 2 studies uncovered evidence that intraclass brand-name drugs restrain launch prices of new drugs, anchoring them to existing benchmarks. Lu and Comanor investigated the pricing trends of all new drugs entering the US market between 1978 and 1987 [15]. Using multivariable modeling, they reported a 38% decrease in the ratio of a new drug’s launch price to the average class price when the number of brand-name drugs in the class increased from 1 to 2, and a 19% decrease when the number increased from 2 to 3.

Howard and colleagues studied cancer drugs entering the market from 1995 to 2013 [23]. After controlling for survival benefits and inflation, they reported a 10% annual increase in per-treatment launch prices. Such a finding, the authors noted, was consistent with reference price models of demand, in which “consumers’ purchase decisions […] depend on a pricing anchor, or reference price, rather than some internal comparison or price and willingness-to-pay” [23].

### Relative quality and marketing as effect modifiers of brand–brand competition

Three investigations found evidence that relative effectiveness and safety (“relative quality”) modified the effect of brand–brand competition on new drug launch prices. Using a then-existing FDA grading scheme for prioritizing the review of new drugs (Grade A: important therapeutic gain, Grade B: modest therapeutic gain, Grade C: little or no therapeutic gain), Lu and Comanor found that launch prices of Grade A and B drugs were on average 77% and 79% higher than existing brand-name drugs within the same class; by contrast, the average launch price of Grade C drugs was 51% lower [15]. Over an 8-year period on the market, the average prices of Grade A and B drugs decreased by 14% and 12%, whereas the average price of Grade C drugs increased by 30%. The authors concluded that manufacturers of therapeutically innovative products used a “skimming” strategy, initially pricing high to signal superior quality and then lowering over time, whereas manufacturers of noninnovative products used a “penetration” strategy, initially pricing low to capture market share and then increasing over time.

Similar findings were reported in studies of H2 blockers and cancer drugs. Berndt and colleagues observed that ranitidine, a drug with a more favorable side-effect profile than cimetidine, commanded a 56% price premium at launch [17]. However, unlike the average price of Grade A drugs in Lu and Comanor’s investigation, the price of ranitidine did not fall over time. Howard and colleagues subsequently estimated that each life-year gained from a new cancer drug was associated with a $75,000 increase in its per-treatment price [22].

Three studies also found evidence that marketing mediated the impact of brand–brand competition on intraclass drug prices. Berndt and colleagues [17] reported that H2 blocker marketing was market expanding when rival products were available, enabling prices to rise from increased demand. Evaluating 360 drugs between 1994 and 2003, de Frutos and colleagues [16] found that higher quality drugs—defined in part based on whether they received “priority” or “standard” review—were more heavily marketed and that marketing was associated with higher prices for all drugs in the class, not just the marketed drug. Finally, Rizzo [18] reported that marketing lowered price sensitivity among 4 different classes of antihypertensive drugs: angiotensin-converting enzyme inhibitors, beta blockers, calcium-channel blockers, and diuretics.

## Discussion

In our systematic literature review, we found no studies that show that brand–brand competition lowers list prices of existing drugs within a class. However, we found evidence that brand–brand competition may anchor the list prices of new drugs below what they would be in the absence of such competition. We also found that the effect of brand–brand competition on drug prices is likely modified by relative drug quality and the extent of marketing, with safer or more effective new drugs commanding higher prices and greater marketing associated with higher intraclass prices.

These findings underscore some distinctive features of the US pharmaceutical market. In a truly competitive market, introduction of similar products should lower prices of previously available products. One reason that the US does not follow this pattern is that physicians primarily determine what drug is prescribed and rarely have direct incentives to select the most cost-effective treatment. The Federal Trade Commission captured this conundrum in a 1979 report, noting that “the forces of competition do not work well in a market where the consumer who pays does not choose and the physician who chooses does not pay” [25]. Physicians are also often unaware of the absolute or relative prices of drugs [26].

Additionally, unlike consumers in more competitive markets, payers in the US pharmaceutical market may face legal limits on their flexibility to negotiate prices. In particular, coverage mandates are prevalent in the US and hinder payers from capitalizing on new brand-name market entrants to achieve lower prices. For example, Medicaid—the federal- and state-funded assistance program for low-income patients—must cover virtually all FDA-approved drugs in exchange for receiving guaranteed rebates equivalent to the best price that brand-name manufacturers get in the private market [27]. Similarly, Medicare Part D plans are required to cover all drugs within 6 protected drug classes, including cancer drugs [28], a field in which numerous follow-on brand-name drugs have been marketed in recent years. Further complicating the cancer drug market, some states have laws requiring payers to cover off-label uses of cancer drugs listed in national compendia [29].

The structure of the US pharmaceutical market may also work against the prospect of brand–brand competition being able to lower list prices. For example, PBMs have emerged as an intermediary to help negotiate drug prices on behalf of public and private payors. But in contracts with insurers, some PBMs have arranged to retain a portion of the rebates they receive from a drug manufacturer. This arrangement can encourage PBMs to accept high list prices because the size of the rebate—and thus the PBM-retained portion of the rebate—would be larger, assuming that PBMs could negotiate the same net price [30]. Promoting brand–brand competition therefore is unlikely to lower list prices without other structural changes to the US pharmaceutical market.

Additional possible explanations for our findings include the limited information available on new drugs and oligopoly dynamics. First, in the absence of comparative safety and effectiveness data, it is possible that higher prices are perceived by some actors as a sign of quality [31,32]. In such situations, brand-name drugs may exhibit properties of Veblen goods, in which higher prices are associated with increasing demand [33], incentivizing ever-higher pricing. Second, when there are a limited number of firms operating in a market—as is often the case for drugs indicated for the same condition—they sometimes engage in tacit collusion to maintain or increase their prices [34], for example, by following the pricing strategy of the leading firm.

The findings of our review raise concern about the economic consequences of pharmaceutical marketing. In 2016, the pharmaceutical industry spent $6.1 billion on direct-to-consumer advertising and likely more than 5 times as much on physician marketing [35,36]. Such promotion may help some patients become aware of new treatments, but a wealth of research has shown that marketing also drives overuse of expensive brand-name drugs [37]. Because marketing may also force patients to pay more for their medications, US policymakers may need to reexamine the extent and manner to which it can take place and explore effective ways to counter its impact [38].

Our review may also be relevant for the emerging US biosimilar market. Biosimilars are versions of originator biologic drugs made by different manufacturers. They are analogous to generic drugs, versions of originator small-molecule drugs made by different manufacturers, but given the greater size and complexity of biologics relative to small molecules, biosimilars are not considered interchangeable without meeting heightened testing standards, which the FDA has only recently begun to define. Accordingly, pharmacists may not automatically substitute prescriptions for biosimilars at this time. Depending on the policies that Congress, the states, and the FDA adopt, biosimilar competition may more closely resemble brand–brand competition than brand–generic competition [39,40].

Three limitations of our review should be noted. First, the results are based on only 10 rigorous empirical studies that met our entry criteria. Within the cohort, the included studies assessed only list prices, except for the investigation by San-Juan-Rodriguez and colleagues [22]. Net prices may be more impacted by brand–brand competition, which may partially explain a seemingly growing difference between net and list prices [41]. However, net prices are considered proprietary trade secrets. List prices remain an important measure of affordability because they influence co-insurance and deductible payments as well as charges for uninsured patients [42].

Second, the assessment periods for about half of the studies in our review were over 2 decades old. Nevertheless, comparable findings were reported by more recent investigations. For example, Hartung and colleagues [21], San-Juan-Rodriguez and colleagues [22], and Gordon and colleagues [24] each reported that new drug entry was not associated with price reductions of existing intraclass brand-name products, which mirrored earlier findings by Berndt and colleagues [17], Bokhari and Fournier [20], and Wiggins and Maness [19]. Similarly, Howard and colleagues [23] observed an anchoring effect of existing intraclass brand-name products on new drug launch prices, which had been previously reported by Lu and Comanor [15]. Regarding drug marketing, we are aware of no evidence that its apparent role in mediating brand–brand competition has changed. Between 1997 and 2016, manufacturer spending on pharmaceutical marketing to physicians increased from $15.6 billion to $20.3 billion, while direct-to-consumer advertising expenditures grew from $1.3 billion to $6 billion [43], suggesting that marketing may now play an even larger role in market expansion.

Finally, our investigation was limited to the US market. A similar review of prescription drug pricing dynamics outside of the US may find different results, because many other high-income countries use variations of reference pricing, in which the prices of new drugs approved in an existing class are tied to the lowest price of the drug in the class unless they have substantial clinical differences [44]. In the case of Canada, however, Lexchin reported similar findings to those uncovered by our systematic review [45].

## Conclusion

Our systematic review found no evidence that brand–brand competition lowers list prices in the US market. While more research is needed to identify whether there are specific situations in which such competition may be impactful, structural reforms are ultimately needed to address the rising price of prescription drugs in the US.

## Supporting information

S1 Table PRISMA 2009 checklist. PRISMA, Preferred Reporting Items for Systematic Reviews and Meta-Analysis.
(DOC)

S1 Text Study protocol. (DOCX)

## Abbreviations

CML: chronic myeloid leukemia
CMS: Centers for Medicare and Medicaid Services
DHHS: Department of Health and Human Services
FDA: Food and Drug Administration
H2: histamine-2
MeSH: medical subject heading
PBM: pharmacy benefits manager
PCSK9: proprotein convertase subtilsin-kexin type 9
PRISMA: Preferred Reporting Items for Systematic Reviews and Meta-Analysis
TNF: tumor necrosis factor
