# Supplementary material for: Competition and price among brand-name drugs in the same class: A systematic review of the evidence
Source: PLoS Med. 2019 Jul 30;16(7):e1002872. doi: 10.1371/journal.pmed.1002872 (PMC6667132; doi:10.1371/journal.pmed.1002872)
Supplement: S1 Text — (DOCX) [file pmed.1002872.s002.docx]

**Competition and Price among Brand-Name Drugs in the Same Class:**

**A Systematic Review of the Evidence**

**Study Protocol**

Original

1. A.S. and M.N. develop search terms for PubMed and EconLit
2. J.D. or M.Z. searches PubMed using search terms and makes a list of identified articles
   1. Search Terms: “‘Drug Costs’ [MeSH] AND (‘Economics, Pharmaceutical’ [Mesh] OR ‘Economic Competition’ [MeSH]) AND ‘United States’ [MeSH]”
   2. Date restriction: 1990 and onward
   3. Language restriction: English language articles only
3. J.D. or M.Z. searches EconLit using search terms and makes a list of identified articles
   1. Search Terms: “‘Drug’ AND ‘Price’ AND (‘Competition’ OR ‘Determinants’ OR ‘Factors’) AND (‘United States’ OR ‘US’)”
   2. Date restriction: 1990 and onward
   3. Language restriction: English language articles only
4. A.S. conducts general Internet search to see if relevant studies excluded
5. J.D. and M.Z. review abstracts of all identified articles and code as topical or not topical
6. A.S. reviews selections, scans full-text of inconsistently scored abstracts, and decides whether topical or not-topical
7. A.S. constructs a final “reasons for exclusion” list
8. M.Z. codes non-topical articles based on reasons for exclusion list
9. J.D. or M.Z. takes notes on selected articles
   1. Objective
   2. Methods
   3. Results
   4. Interpretation
10. A.S. and M.N. review notes and articles

Update

1. A.S. and M.Z. develop new non-MeSH search terms for PubMed
2. M.Z. searches PubMed using non-MeSH search terms and makes list of identified articles
   1. Search Terms: “(‘Drug Costs’ OR ‘Drug Prices’ OR ‘Cost Changes’ OR ‘Price Changes’) AND (‘Competition’ OR ‘Competitors’)”
   2. Date restriction: February 2018 and onward
   3. Language restriction: English language articles only
3. M.Z. searches EconLit using search terms and makes a list of identified articles
   1. Search Terms: “‘Drug’ AND ‘Price’ AND (‘Competition’ OR ‘Determinants’ OR ‘Factors’) AND (‘United States’ OR ‘US’)”
   2. Date restriction: February 2018 and onward
   3. Language restriction: English language articles only
4. M.Z. and A.S. review all identified articles and code as topical or not topical
5. M.Z. codes non-topical articles based on reasons for exclusion list
6. M.Z. takes notes on selected articles
   1. Objective
   2. Methods
   3. Results
   4. Interpretation
7. A.S. reviews notes and articles

Final Step

1. Team analyzes data and performs qualitative synthesis
